# Gut Microbiota Recovery After Direct-Acting Antiviral Therapy for Chronic Hepatitis C: A Systematic Review and Meta-Analysis

**DOI:** 10.3390/microorganisms14081843

**Published:** 2026-08-19

**Authors:** Jing-Hong Hu, Ming-Ling Chang, Tung-Jung Huang, Yung-Yu Hsieh, Nai-Jen Liu, Kai-Feng Sung, Jui-Hsiang Tang

**Affiliations:** 1 Division of Gastroenterology and Hepatology, Yunlin Chang Gung Memorial Hospital, Yunlin County 638, Taiwan; 2 Division of Gastroenterology and Hepatology, Linkou Chang Gung Memorial Hospital and College of Medicine, Chang Gung University, Taoyuan 333, Taiwan; mlchang8210@gmail.com; 3 Division of Pulmonary and Critical Care Medicine, Yunlin Chang Gung Memorial Hospital, Yunlin County 638, Taiwan; donaldhuang@cgmh.org.tw; 4 Division of Gastroenterology and Hepatology, Chiayi Chang Gung Memorial Hospital, Chiayi County 613, Taiwan; ivan5510@gmail.com; 5 Department of Gastroenterology and Hepatology, Linkou Chang Gung Memorial Hospital, Taoyuan 333, Taiwan; launaijn.tw@yahoo.com.tw (N.-J.L.); h12153@adm.cgmh.org.tw (K.-F.S.); 6 Division of Gastroenterology and Hepatology, Department of Internal Medicine, Jen-Ai Hospital, Dali Branch, Taichung 412, Taiwan; ercp.tw@msa.hinet.net

**Keywords:** hepatitis C virus, direct-acting antivirals, gut microbiota recovery, dysbiosis, gut–liver axis, microbial metabolites, systematic review, meta-analysis

## Abstract

Direct-acting antivirals (DAAs) cure most chronic hepatitis C virus (HCV) infections, yet whether the gut microbiota returns toward a healthy state after viral clearance remains uncertain. We systematically reviewed DAA-era adult HCV studies using sequencing-based fecal microbiota assessment (PROSPERO CRD420261374682). Longitudinal alpha-diversity change was pooled by REML random-effects meta-analysis with Hartung–Knapp adjustment, and compositional/functional findings were synthesized narratively. Seven studies met qualitative criteria and five longitudinal reports were extractable. Because participant overlap between two Thai reports could not be excluded, the primary conservative non-overlap analysis of four reports (180 paired observations) gave Hedges’ g = 0.13 (95% CI −0.53 to 0.80; I^2^ ≈ 89%); the five-report sensitivity estimate was directionally positive but imprecise (g = 0.35, 95% CI −0.38 to 1.07). Recovery concentrated in richness (Chao1), whereas evenness-weighted diversity (Shannon, Hill) barely moved; beneficial taxa such as *Faecalibacterium* and *Blautia* increased after SVR. These findings are consistent with uneven, richness-led microbial recovery, but the overall certainty of the pooled evidence is very low (GRADE); functional and clinical recovery remain insufficiently characterized and undemonstrated.

## 1. Introduction

Direct-acting antivirals have transformed chronic hepatitis C virus (HCV) infection into a curable disease, with sustained virological response (SVR) rates exceeding 95% in most treated populations [1,2]. Virological cure is conventionally defined as a sustained virological response (SVR)—undetectable serum HCV RNA at least 12 weeks after the end of therapy (SVR12). Viral eradication, however, does not by itself guarantee biological recovery. Residual fibrosis, metabolic dysfunction, immune remodeling, and hepatocellular carcinoma (HCC) risk can persist after SVR, particularly in patients treated at advanced stages of liver disease. Although SVR markedly reduces HCC risk, it does not eliminate it: recent syntheses report that post-SVR HCC incidence remains approximately 1–2 per 100 person-years in patients with cirrhosis, with substantially lower rates in those without cirrhosis [3,4,5,6,7]. Major societies therefore agree on continued HCC surveillance after SVR for patients with cirrhosis, whereas recommendations for pre-SVR F3 fibrosis differ between EASL and AASLD [3,4,6,7]. An unresolved question is whether the gut ecosystem, which is disturbed during chronic infection, returns toward a healthy state once the virus is cleared.

The gut microbiota is increasingly recognized as a component of liver health through the gut–liver axis. Portal venous flow, bile acids, microbial metabolites, intestinal barrier function, and host immune signaling link the intestinal ecosystem to hepatic inflammation and fibrogenesis [8,9]. Chronic HCV infection has been associated with dysbiosis—a departure from the compositional and functional balance of a healthy gut community—together with altered microbial diversity, depletion of short-chain-fatty-acid-producing taxa, and enrichment of potentially inflammatory organisms [10,11,12]. If these disturbances do not resolve after cure, the intestine may remain a source of inflammatory and metabolic signaling relevant to residual liver risk, which makes microbial recovery a biologically meaningful and clinically motivated endpoint.

Whether DAA-induced eradication is followed by meaningful microbiota recovery—here defined as movement of the post-treatment gut community back toward the taxonomic composition, ecological diversity, and functional profile seen in uninfected or healthy controls, recognizing that these dimensions—and any downstream clinical recovery—may normalize at different rates—is unsettled. Some cohorts report improved alpha diversity or enrichment of beneficial taxa after SVR, especially with milder fibrosis or longer follow-up [13,14]; others find little change in overall diversity soon after clearance [15,16,17]. Newer work links microbial recovery to bile-acid remodeling and broader gut–liver axis restoration [18].

Earlier syntheses have been largely narrative or have emphasized cross-sectional dysbiosis during active infection rather than quantitatively pooling longitudinal recovery after DAA-induced cure; a pre-registered quantitative synthesis focused specifically on post-SVR recovery has therefore been lacking. We therefore asked: to what extent does the gut microbiota recover after successful HCV eradication? Alpha diversity served as the primary quantitative endpoint because it was the most consistently extractable longitudinal metric. Taxonomic composition and functional findings were synthesized narratively to provide a cautious biological interpretation.

## 2. Materials and Methods

### 2.1. Search Strategy and Reporting Standard

This review followed PRISMA 2020 reporting principles [19]. We searched PubMed, Embase, Web of Science, and the Cochrane Library from database inception to 16 April 2026 using combinations of terms related to hepatitis C, direct-acting antivirals, gut microbiota, microbiome, and dysbiosis. Database-specific search strings are provided in Table S1.

The protocol was registered in PROSPERO (CRD420261374682; search date, 16 April 2026; registration, 20 April 2026). Registration followed the search, reflecting administrative processing time, but preceded any data extraction or quantitative analysis. Because the registered protocol also listed glycemic and cardiovascular outcomes, this manuscript reports the gut-microbiota component of that broader review; the other outcomes are reported separately and were not analyzed here.

### 2.2. Eligibility Criteria

We included original studies of adults with chronic HCV treated with DAAs in which fecal microbiota was assessed using 16S rRNA sequencing or equivalent sequencing-based methods. Eligible designs were longitudinal pre/post-treatment cohorts and cross-sectional studies comparing treated or post-SVR patients with active-HCV and/or healthy controls. We excluded interferon-based treatment studies—whose immunomodulatory and microbiota-altering effects could confound the evaluation of DAA-specific effects—as well as case reports, conference abstracts without analyzable data, editorials, and reviews.

After full-text assessment, review articles were excluded from both qualitative and quantitative analyses. Qualitative synthesis included original DAA-era studies reporting microbiota composition or diversity that could inform post-treatment recovery. Studies restricted to participants selected by a post-SVR clinical outcome (e.g., prevalent HCC) and lacking a pretreatment or active-HCV comparator were not used to estimate recovery, although they could be cited as contextual evidence in the Discussion. Quantitative meta-analysis was restricted to longitudinal cohorts with extractable alpha-diversity information before and after DAA therapy.

### 2.3. Data Extraction and Risk-of-Bias Assessment

Two investigators independently rechecked each full text and extracted study design, country, analyzable cohort size, fibrosis stage, DAA regimen, sequencing target region, alpha-diversity metric, follow-up time point, and microbiota findings. When exact numerical summaries were unavailable, values were reconstructed using WebPlotDigitizer (version 4.7); each figure was digitized independently by two investigators, discrepancies were resolved by consensus, and extracted values were verified against the published plots before quantitative synthesis. Reconstructed estimates were treated as approximate and carried into the certainty assessment.

Methodological quality was assessed using the Newcastle–Ottawa Scale (NOS) [20]. Because the included studies were small observational microbiome cohorts, NOS assessments were interpreted descriptively rather than as exclusion criteria (Table S2). The NOS rates three domains—selection (up to four stars), comparability (up to two stars), and outcome/exposure ascertainment (up to three stars)—for a maximum of nine stars, with higher scores indicating lower risk of bias; per-study domain scores are reported in Table S2.

### 2.4. Quantitative Synthesis

The primary quantitative endpoint was longitudinal change in alpha diversity after DAA therapy. Because studies did not report a uniform metric, the synthesis used the standardized mean change with Hedges’ correction. Standardized mean change permits pooling across studies that report conceptually related but non-identical continuous scales, as recommended for such meta-analyses [21,22]. Because richness indices (e.g., Chao1) and diversity/evenness indices (e.g., Shannon, Hill numbers) quantify different ecological properties, this pooling was pre-specified as a limitation and complemented by a metric-stratified descriptive analysis (Figure S2; Table S3). Pooling was performed only to summarize standardized longitudinal change rather than to imply biological equivalence among ecological indices. Where medians, ranges, or interquartile ranges were reported, approximate means and standard deviations were reconstructed using established methods [23,24]. A within-subject correlation of r = 0.50 was assumed for change-score standardization—the conventional value when the pre/post correlation is unreported, and consistent with previous paired pre/post meta-analyses—with r = 0.25 and r = 0.75 examined in sensitivity analyses (Table S4).

Random-effects meta-analysis used REML with Hartung–Knapp adjustment as the primary model. Between-study heterogeneity was quantified with τ^2^, the Q statistic, and I^2^. Because the Hartung–Knapp method uses a t-distribution with k − 1 degrees of freedom, confidence intervals are necessarily wide when few studies are available (3 degrees of freedom for the primary four-report analysis). Prediction intervals and leave-one-out analyses evaluated robustness. Because two Thai reports from the same group could not be confirmed as independent from published data alone, a conservative non-overlap analysis retained the longer-follow-up report for the primary interpretation and treated the other as sensitivity evidence (Figure S1). DerSimonian–Laird pooling was retained only as a sensitivity analysis. Publication bias was not formally assessed because fewer than 10 studies were available [25]. Meta-regression and network meta-analysis were not performed because the number of eligible studies fell below accepted thresholds and comparative DAA-regimen data were insufficient. We used REML random-effects meta-analysis with Hartung–Knapp adjustment as the primary framework and, throughout, distinguished measured compositional change from the functional and clinical recovery that current data cannot yet establish. The certainty of the pooled alpha-diversity outcome was appraised with the GRADE framework, starting at low certainty because all contributing studies were observational and downgrading further for risk of bias, inconsistency, indirectness, and imprecision (the reliance on figure-assisted reconstruction for several estimates contributed to the risk-of-bias downgrade); the full domain-by-domain assessment is provided in the Supplementary Materials.

All meta-analyses were conducted using R version 4.6.0 (R Foundation for Statistical Computing, Vienna, Austria) with the metafor package version 5.0-1.

## 3. Results

### 3.1. Study Selection

**Figure 1:**
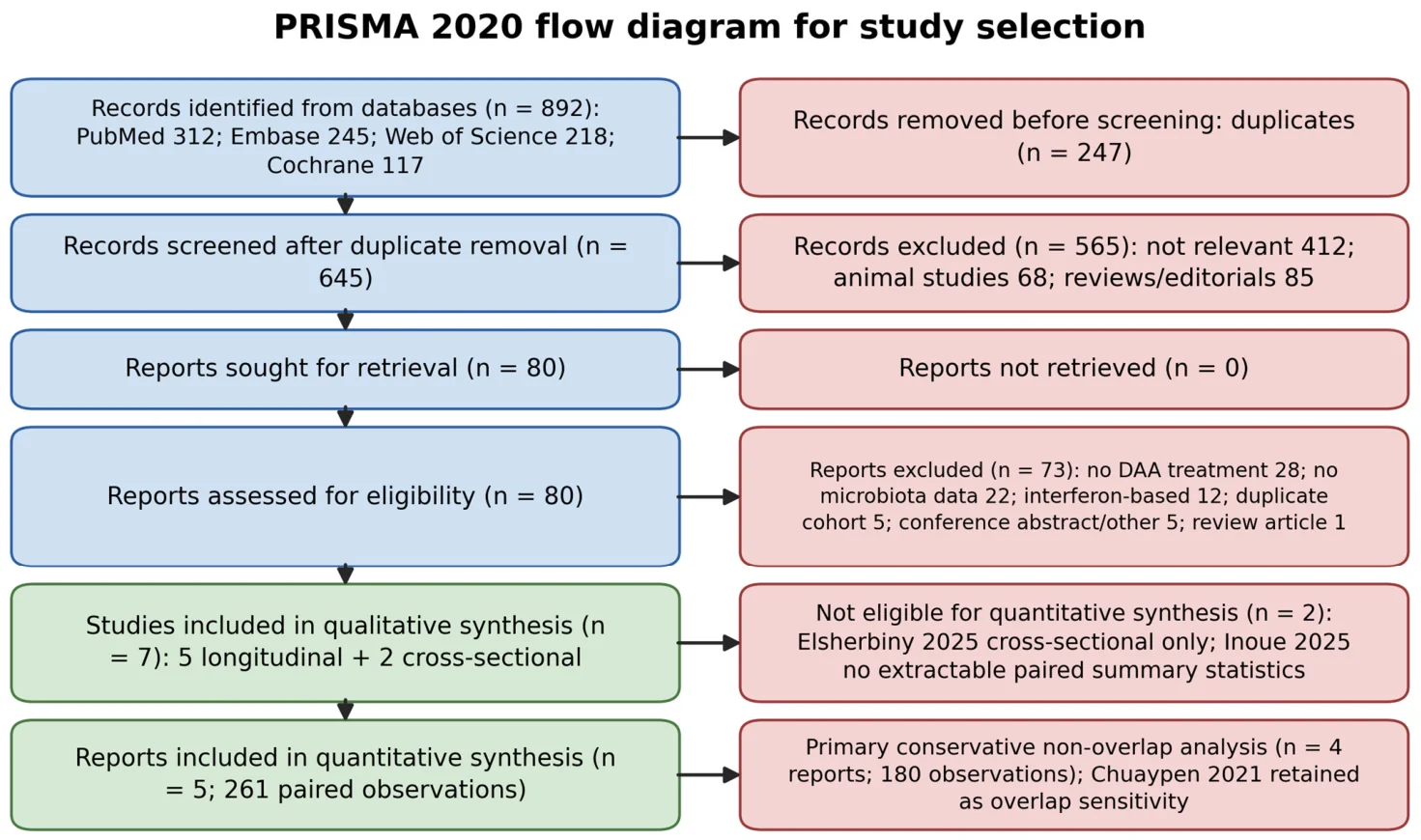
PRISMA 2020 flow diagram for study selection. Five reports entered quantitative synthesis; the primary conservative (non-overlap) analysis used four reports (180 paired observations), with Chuaypen 2021 [13] retained only for the five-report sensitivity analysis.

The database search yielded 892 records. After duplicate removal, 645 titles and abstracts were screened, and 80 full-text articles were assessed for eligibility. Seven original studies met qualitative eligibility criteria, and five longitudinal cohorts provided sufficient alpha-diversity data for quantitative synthesis (Figure 1).

### 3.2. Included Studies and Cohort Accounting

The final evidence base comprised studies from Egypt, Japan, Taiwan, Thailand, and Switzerland. Designs included longitudinal pre/post-treatment cohorts and cross-sectional comparisons of treated, untreated, relapsed, and healthy-control groups. Cohorts differed in fibrosis stage, follow-up duration, sequencing target region, and alpha-diversity metric (Table S5).

Five longitudinal reports provided extractable data for quantitative synthesis. The most frequently available alpha-diversity metrics were Shannon, Chao1, Simpson, and observed species. Because the two Thai reports originated from the same research group and institution and applied similar inclusion criteria—so that participant continuation into the longer-follow-up report could not be excluded—they could not be confirmed as independent at the participant level; the primary analysis therefore used four independent report-level estimates and retained the five-report analysis as a sensitivity analysis. Cross-sectional and functional findings were kept for qualitative interpretation but not pooled.

### 3.3. Primary Quantitative Outcome: Longitudinal Alpha-Diversity Change

**Figure 2:**
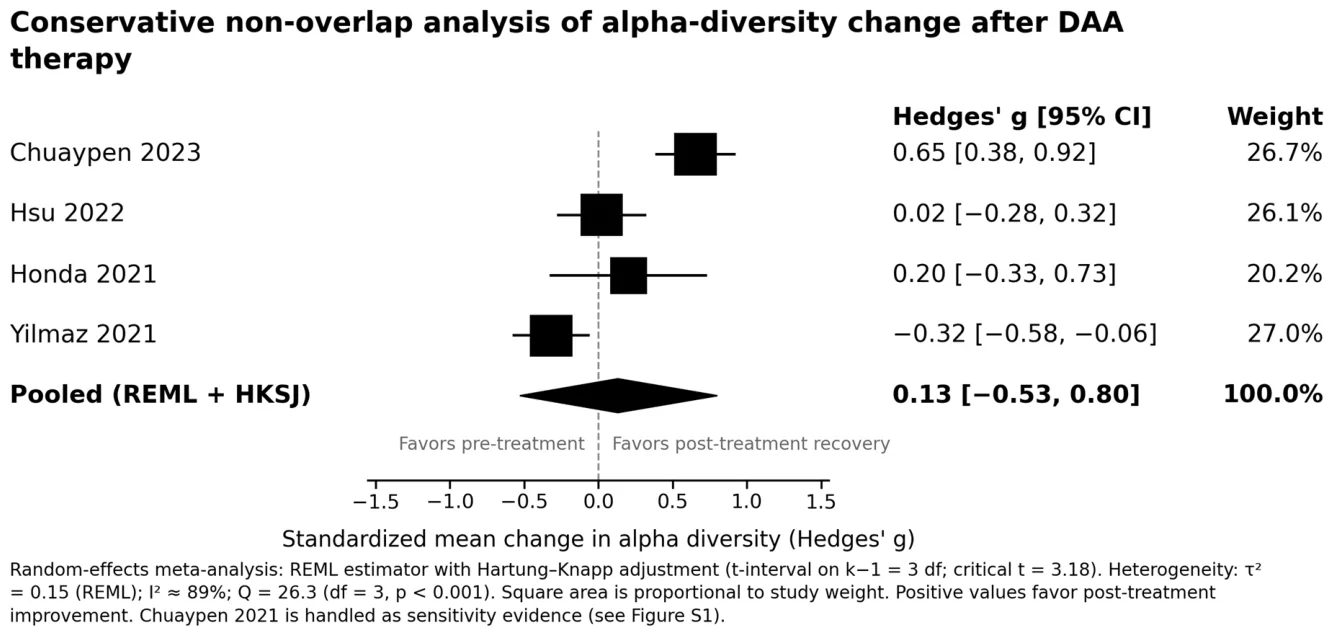
Conservative non-overlap forest plot of standardized longitudinal alpha-diversity change after DAA therapy (REML random-effects meta-analysis with Hartung–Knapp adjustment). Square area is proportional to random-effects study weight. Positive values favor post-treatment improvement. The studies included in the primary analysis were Chuaypen et al. [14], Hsu et al. [15], Honda et al. [16], and Yilmaz et al. [17].

In the conservative non-overlap REML + Hartung–Knapp analysis (four reports; 180 paired observations), the pooled standardized mean change was Hedges’ g = 0.13 (95% CI −0.53 to 0.80; τ^2^ = 0.15 [REML]; I^2^ ≈ 89%; Q = 26.3, df = 3, *p* < 0.001; Figure 2).

The five-report sensitivity analysis including both Thai publications yielded g = 0.35 (95% CI −0.38 to 1.07; τ^2^ = 0.32), with an approximate prediction interval of −1.3 to 2.0 (Figure S1). A DerSimonian–Laird sensitivity analysis for the same dataset gave g = 0.35 (95% CI −0.21 to 0.91; τ^2^ = 0.38; I^2^ = 94.0%).

The largest per-study estimates arose in the two Thai reports, both of which used Chao1 richness-based metrics. When the shorter-follow-up Thai report was omitted, the pooled estimate moved toward the null. Shannon/Hill-type diversity metrics from Hsu, Honda, and Yilmaz were individually near null (g = 0.02, 0.20, and −0.32, respectively; Figure S2; Table S3). In leave-one-out analyses, the pooled point estimate ranged from 0.13 to 0.52, and every interval included the null (Table S6). Heterogeneity was substantial (I^2^ ≈ 89%); its likely sources—fibrosis stage, follow-up duration, sequencing platform, and population differences—are examined qualitatively in Section 4.7, since formal meta-regression was not feasible.

### 3.4. Secondary Qualitative Synthesis: Taxonomic and Functional Findings

Directional compositional changes were more consistent than diversity changes. SCFA-associated genera such as *Faecalibacterium* and *Blautia* increased after SVR in several cohorts, whereas inflammation-associated or potentially pathogenic taxa—including *Enterobacteriaceae*, *Streptococcus*, and *Staphylococcus* in selected reports—decreased, though not universally. Functional data were sparse: paired shotgun metagenomics, metabolomics, and host immune phenotyping were reported in only a few cohorts.

## 4. Discussion

### 4.1. Principal Findings and Interpretation

This review addresses a central question in hepatitis C care: does the gut microbiota return toward a healthy state once the virus is cleared? Across the available longitudinal data, recovery of overall alpha diversity was modest and uneven. These findings rest on a small evidence base (seven studies; five longitudinal) with substantial heterogeneity and very low GRADE certainty, and should be read as hypothesis-generating throughout. The conservative non-overlap estimate was small, and its interval crossed the null, whereas the larger five-report sensitivity estimate was driven mainly by richness-based Thai data. The magnitude is small: at a pooled g near 0.1–0.4, alpha-diversity change is better suited to generating hypotheses than to serving as a clinical biomarker.

Rather than resting on a claim of primacy, the contribution of this work is to reframe an inconsistent literature. We provide a pre-registered, conservative quantitative estimate, separate richness- from evenness-based signals, and grade the certainty of the pooled outcome. In doing so, the analysis recasts “microbiota recovery” from a single expected effect into a staged, metric-dependent process, and it makes explicit that viral cure and microbial cure are not equivalent.

This distinction carries a clinical rationale. If the microbiota does not fully normalize after SVR, the intestine may keep contributing inflammatory and metabolic signaling through the gut–liver axis, which is plausibly relevant to residual fibrosis, persistent immune activation, altered bile-acid handling, and the HCC risk that remains in some patients after cure [3,4,5,6,7]. Microbial recovery may represent one explanation for why viral cure does not completely abolish downstream risk, even though present data cannot yet confirm these links. Although persistent dysbiosis may contribute to residual liver inflammation and carcinogenic signaling through the gut–liver axis, current evidence remains observational and does not establish a causal relationship between post-SVR microbiota recovery and subsequent HCC development.

### 4.2. Comparison with Previous Studies

The largest recovery signals came from Thai cohorts with longer observation and fibrosis-stratified analyses [13,14]. Because these two reports arose from one research group and participant overlap could not be excluded from published information, we treat the non-overlap analysis as the most defensible primary reading. Short-term studies generally found little change in alpha or beta diversity after DAA therapy [15,16,17]. Honda and colleagues reported no major overall diversity shift, yet observed enrichment of *Faecalibacterium* after eradication, indicating that selected taxa may recover even when community-level indices are stable [16]. Inoue 2025 was retained qualitatively but not pooled because extractable paired alpha-diversity statistics were unavailable [18].

Recent 2025–2026 literature supports a measured reading. A pilot study reported that DAA treatment reshaped gut microbiota profiles in chronic HCV [26]. A highly selected cross-sectional study, published online in 2026, compared diabetic post-SVR patients with and without prevalent HCC and healthy controls; it described persistent dysbiosis, depletion of butyrate-producing *Faecalibacterium*, reduced predicted butyrate synthesis, and increased lipopolysaccharide biosynthesis [27]. Because it lacked a pretreatment or active-HCV comparator, it is used here only as contextual evidence and does not inform longitudinal recovery. Contemporary reviews of HCV dysbiosis, viral-hepatitis metabolomics, and the gut–liver axis similarly emphasize that composition, bile-acid signaling, SCFA production, immune regulation, and clinical outcomes may recover at different rates [28,29,30,31,32,33].

### 4.3. Biological Interpretation and a Staged Recovery Model

**Figure 3:**
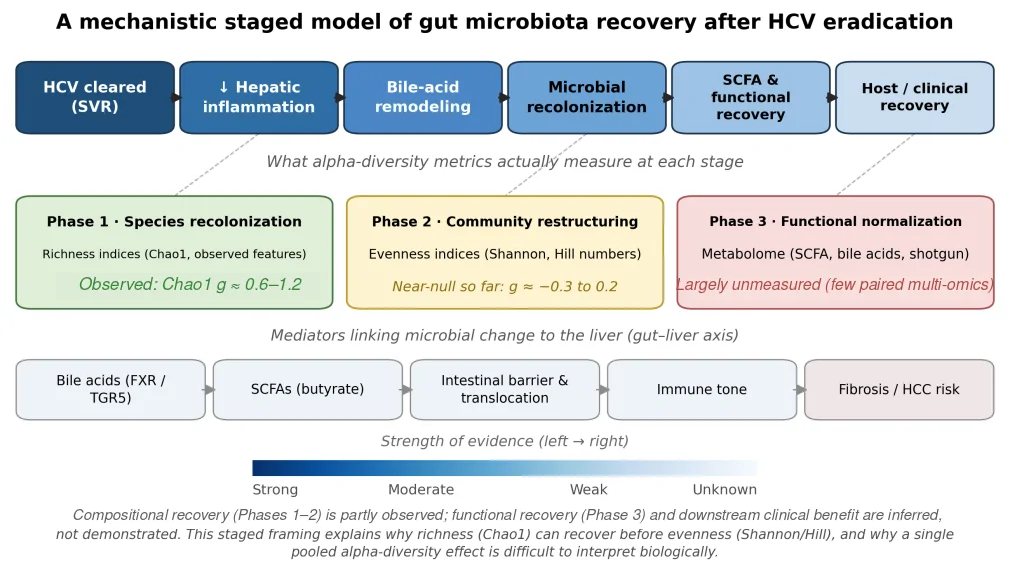
A mechanistic staged model of gut microbiota recovery after HCV eradication. A causal spine (SVR → reduced hepatic inflammation → bile-acid remodeling → microbial recolonization → functional recovery → host/clinical recovery) is mapped to the alpha-diversity metrics that detect each phase and to gut–liver-axis mediators (bile acids, SCFAs, barrier integrity, immune tone). Strength of evidence decreases from left to right; only the earliest stages are supported by current data.

Mechanistically, we hypothesize that viral clearance lowers hepatic inflammation and remodels bile-acid pools, which in turn permits microbial recolonization, gradual functional (short-chain fatty acid and metabolite) normalization, and only then any downstream immune and clinical benefit (Figure 3). Each link in this chain is progressively less certain than the one before it, and only the earliest links are supported by the present data.

These metric-specific patterns fit a hypothesis-generating staged recovery model (Figure 3). In an early phase, previously depleted low-abundance taxa recolonize the gut, raising richness indices such as Chao1 while community dominance is largely preserved. In a second phase, relative abundances re-equilibrate—the change detected by evenness-weighted indices such as Shannon and Hill numbers—and this restructuring appears slower and was not clearly captured within the follow-up windows studied. A third phase, functional normalization of short-chain fatty acids, bile-acid handling, and the wider metabolome, was seldom measured with paired sampling. Reading the data through this model explains why Chao1 recovery can precede Shannon/Hill recovery, and why collapsing both into one standardized effect obscures more than it reveals.

Microbial metabolites are plausible mediators of this sequence. SCFAs, particularly butyrate, support epithelial barrier function and immune regulation [34], and bile acids shape microbial ecology while signaling through FXR and TGR5 to link the microbiome with hepatic metabolism and inflammation [29,35]. Because the included HCV studies rarely paired microbiome profiling with metabolomics, functional recovery is inferred rather than demonstrated.

### 4.4. Cross-Disease Context

It helps to place these findings alongside microbiome recovery in other chronic liver diseases. In metabolic dysfunction-associated steatotic liver disease and alcohol-associated liver disease, dysbiosis is tied to ongoing metabolic or toxic exposure, so microbial improvement tends to track weight loss, metabolic control, or abstinence rather than removal of a single agent. HCV eradication differs in that the causal driver is removed abruptly and completely, which might be expected to allow faster ecological rebound; even so, the present data indicate that community-level recovery is incomplete on the timescales studied. In hepatitis B, where the virus is suppressed rather than eliminated, residual dysbiosis has likewise been described [31,32]. A formal cross-disease meta-analysis is not yet feasible because diversity metrics, follow-up windows, and reporting are not harmonized across these disease-specific literatures, so the comparison is necessarily qualitative.

### 4.5. Clinical Relevance and Precision Microbiome Monitoring

If microbial recovery is uneven, some patients are more informative to follow than others. Recovery appeared weakest or most variable in cohorts with features that plausibly constrain ecological rebound: advanced fibrosis or cirrhosis, diabetes and higher body-mass index, HIV coinfection, and gut-altering exposures such as proton-pump inhibitors and antibiotics. These are candidate effect modifiers and belong in pre-specified strata. We do not advocate microbiome-guided management or microbiome-directed therapies on current evidence; the practical message is narrower—SVR should not be equated with complete biological recovery, and these subgroups mark where longitudinal multi-omic monitoring would most likely detect meaningful signals.

### 4.6. Is Alpha Diversity an Adequate Surrogate?

Alpha diversity is a convenient summary of within-sample ecological structure, but it is not a direct measure of microbial function. Taxonomic diversity may remain stable while metabolomic or other functional layers shift, and similar diversity values can coexist with distinct transcriptional, proteomic, and metabolomic states [36,37]. Diversity indices should therefore be treated as coarse ecological descriptors rather than surrogate clinical endpoints: a null change does not exclude functional recovery, and a positive change does not establish it. This limitation reinforces the hypothesis-generating interpretation of the pooled estimate and supports paired metagenomic, transcriptomic, proteomic, and metabolomic measurements in future longitudinal studies.

### 4.7. Sources of Heterogeneity

Several factors likely drove heterogeneity. Baseline fibrosis stage varied and repeatedly appeared to modify recovery, with milder fibrosis associated with more favorable shifts. Follow-up duration differed substantially, and short-term sampling may miss gradual change. Sequencing regions, bioinformatic pipelines, and diversity metrics also differed, limiting harmonization. Diet, antibiotic or probiotic exposure, proton-pump inhibitor use, diabetes, obesity, alcohol use, HIV coinfection, and geographic population structure were incompletely reported and should be treated as core covariates in future work.

### 4.8. Future Research Priorities

The next generation of HCV microbiome studies should move beyond taxonomic description. Longitudinal cohorts should include paired sampling before treatment and at SVR12, SVR24, and ideally SVR48 or later, integrating shotgun metagenomics, fecal and serum metabolomics, bile-acid profiling, barrier biomarkers, and host immune phenotyping with clinical outcomes. To make such studies poolable, we propose a minimum reporting checklist for HCV microbiome recovery studies (Checklist S1), designed as a domain-specific companion to community standards such as STORMS and MIxS [38,39], so that recovery trajectories can be compared across populations with less residual heterogeneity. In brief, the checklist asks future studies to report standardized sampling time points (baseline, end of treatment, SVR12, SVR24, and preferably SVR48); the sequencing target region or shotgun platform and the bioinformatic pipeline; a common set of diversity metrics (Shannon, Chao1 or observed features, and a beta-diversity distance); per-time-point effect-size inputs (mean, SD, and n, or paired change with the pre/post correlation); major medication exposures (antibiotics, probiotics, prebiotics, proton-pump inhibitors, and metformin); host effect modifiers (fibrosis stage, diabetes, BMI, alcohol use, HIV status, and diet); functional profiling (shotgun metagenomics, metabolomics, and bile-acid measurement) where feasible; and public deposition of raw sequence data and analysis code. Reporting these items would allow standardized mean-change pooling without figure reconstruction and reduce the between-study heterogeneity observed here.

### 4.9. Limitations

Only five longitudinal reports contributed extractable data, and potential participant overlap between two Thai reports could not be excluded from published information; the conservative non-overlap analysis provides the safest primary reading. Because individual participant data were unavailable, multilevel or cluster-robust variance estimation could not be applied to formally model the possible overlap, so the non-overlap analysis was used instead. Studies reported different alpha-diversity metrics, and several estimates required figure-assisted reconstruction. Follow-up duration, fibrosis stage, and sequencing pipelines varied. Publication bias, meta-regression, and network meta-analysis were not appropriate given the small number of studies and the absence of comparative DAA-regimen data. Most importantly, this review evaluates compositional recovery, for which the GRADE certainty of the pooled alpha-diversity outcome is very low; it cannot establish full normalization, metabolomic recovery, immune restoration, reduced HCC risk, or causal links between microbiota change and clinical outcomes.

### 4.10. Evidence Cascade

**Figure 4:**
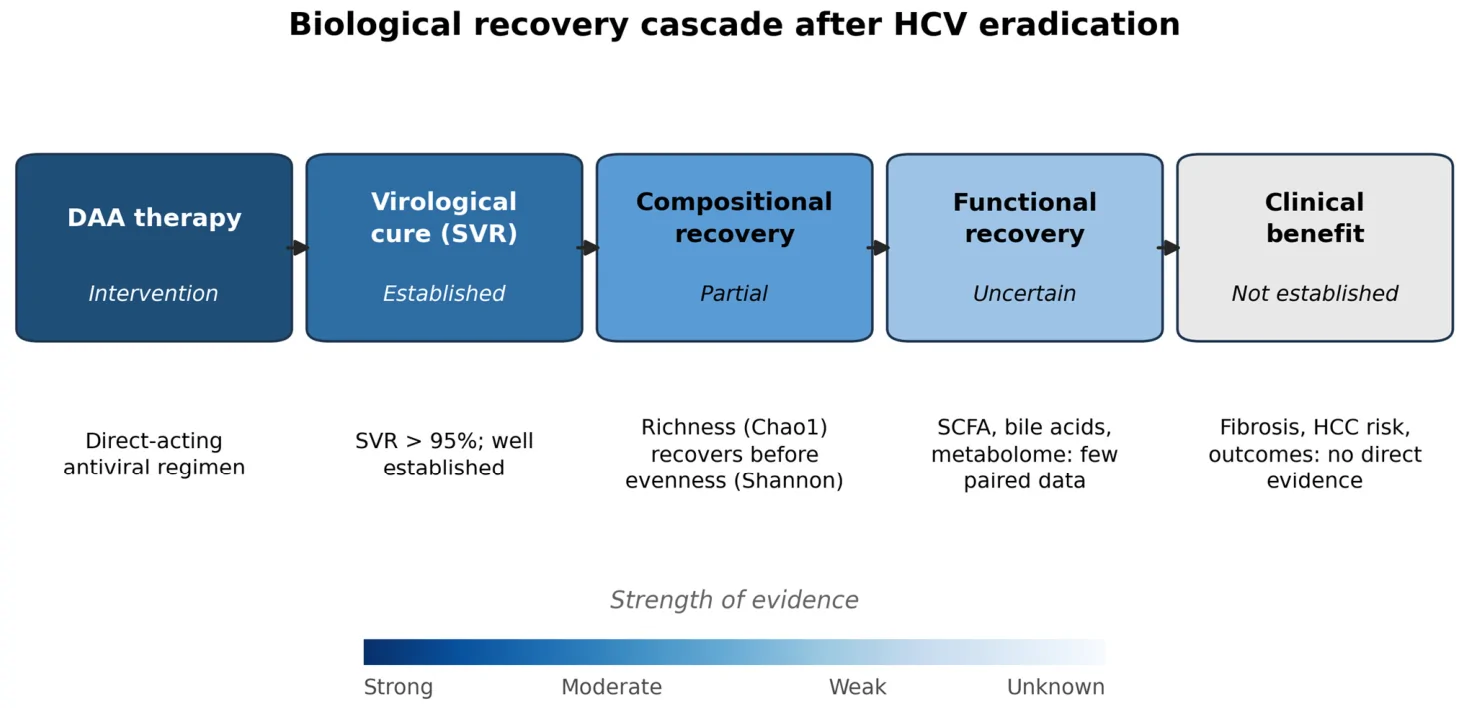
Biological recovery cascade after HCV eradication. The strength of evidence decreases from left (established viral cure) to right (unknown clinical benefit); compositional recovery is partial and richness-led, and functional and clinical stages remain undemonstrated.

Figure 4 summarizes the evidence as a biological cascade from intervention to clinical benefit, with a corresponding gradient in the strength of evidence. Virological cure is well established, compositional recovery has limited support, and functional and clinical recovery are not yet established.

## 5. Conclusions

Gut microbiota recovery after DAA therapy appears partial and richness-led, with quantitative evidence of very low certainty according to GRADE that attenuates once potentially overlapping Thai reports and diversity-metric differences are handled conservatively. Functional recovery of microbial metabolism and the long-term clinical meaning of these changes are not established. Viral eradication should therefore not be interpreted as biological normalization, and longitudinal multi-omic studies—read through a staged recovery framework—are needed to determine whether microbial recovery after HCV eradication translates into improved host and liver health.

## Data Availability

No new individual-level dataset was generated. Extracted and reconstructed study-level data are summarized in the manuscript and Supplementary Materials.

## Supplementary Materials

The following supporting information can be downloaded at: https://www.mdpi.com/article/10.3390/microorganisms14081843/s1. Table S1: Database search strategy; Table S2: Study quality (Newcastle–Ottawa Scale); Table S3: Metric-stratified alpha-diversity estimates; Table S4: Quantitative extraction sheet; Table S5: Characteristics of included studies; Table S6: Leave-one-out sensitivity; Checklist S1: Minimum reporting checklist for future HCV microbiome recovery studies; Figure S1: Five-report sensitivity-analysis forest plot; Figure S2: Metric-stratified alpha-diversity display; GRADE certainty assessment (pooled alpha-diversity outcome).

## Abbreviations

CI	confidence interval
DAA	direct-acting antiviral
GRADE	Grading of Recommendations, Assessment, Development, and Evaluation
HCC	hepatocellular carcinoma
HCV	hepatitis C virus
HIV	human immunodeficiency virus
NOS	Newcastle–Ottawa Scale
PRISMA	Preferred Reporting Items for Systematic Reviews and Meta-Analyses
PROSPERO	International Prospective Register of Systematic Reviews
REML	restricted maximum likelihood
SCFA	short-chain fatty acid
SVR	sustained virological response
