# Supplementary material for: Gut Microbiota Recovery After Direct-Acting Antiviral Therapy for Chronic Hepatitis C: A Systematic Review and Meta-Analysis
Source: Microorganisms. 2026 Aug 19;14(8):1843. doi: 10.3390/microorganisms14081843 (PMC13515309; doi:10.3390/microorganisms14081843)
Supplement: Supplementary file 1 [file microorganisms-14-01843-s001.zip › microorganisms-4465220-supplementary.pdf]

Supplementary Materials

Gut Microbiota Recovery after Direct-Acting Antiviral Therapy for Chronic Hepatitis C: A Systematic Review and Meta-Analysis

Grading of Recommendations Assessment, Development and Evaluation (GRADE) certainty assessment (pooled alpha-diversity outcome): Very low

Starting certainty was low because all contributing studies were observational microbiome cohorts. The rating was further downgraded for risk of bias/measurement limitations (figure-assisted extraction in several cohorts), inconsistency (very high heterogeneity under conventional random-effects estimation), indirectness (mixed alpha-diversity metrics), and imprecision (Hartung–Knapp confidence interval crossing the null). Interpretation: the pooled estimate should be considered hypothesis-generating rather than confirmatory.

Table S1. Database search strategy (executed 16 April 2026)

Database	Search string
PubMed	(“Hepatitis C”[Mesh] OR HCV[Title/Abstract] OR “hepatitis C”[Title/Abstract]) AND (“direct-acting antiviral”[Title/Abstract] OR DAA[Title/Abstract] OR sofosbuvir[Title/Abstract] OR glecaprevir[Title/Abstract] OR pibrentasvir[Title/Abstract]) AND (“gastrointestinal microbiome”[Mesh] OR “gut microbiota”[Title/Abstract] OR microbiome[Title/Abstract] OR dysbiosis[Title/Abstract])
Embase	('hepatitis c'/exp OR hcv:ab,ti OR 'hepatitis c':ab,ti) AND ('direct acting antiviral':ab,ti OR daa:ab,ti OR sofosbuvir:ab,ti OR glecaprevir:ab,ti OR pibrentasvir:ab,ti) AND ('gut microbiota':ab,ti OR microbiome:ab,ti OR dysbiosis:ab,ti)
Web of Science	TS=(“hepatitis C” OR HCV) AND TS=(“direct-acting antiviral” OR DAA OR sofosbuvir OR glecaprevir OR pibrentasvir) AND TS=(“gut microbiota” OR microbiome OR dysbiosis)
Cochrane Library	(“Hepatitis C” OR HCV) AND (“direct-acting antiviral” OR DAA) AND (“gut microbiota” OR microbiome OR dysbiosis)

Table S2. Study quality (Newcastle–Ottawa Scale)

Study	Selection (max 4)	Comparability (max 2)	Outcome/Exposure (max 3)	Total (max 9)
Elsherbiny 2025	3	2	2	7
Chuaypen 2023	4	1	3	8
Inoue 2025	4	1	3	8
Hsu 2022	3	1	3	7
Honda 2021	3	0	3	6
Yilmaz 2021	3	1	3	7
Chuaypen 2021	4	1	3	8

Table S3. Metric-stratified alpha-diversity estimates (descriptive)

Report	Alpha-diversity metric	Ecological dimension	Hedges’ g	Analysis set
Chuaypen 2023	Chao1	Richness	0.65	Primary + exploratory
Chuaypen 2021	Chao1	Richness	1.17	Exploratory only
Hsu 2022	Shannon/Hill	Diversity/evenness	0.02	Primary + exploratory
Honda 2021	Shannon/Hill	Diversity/evenness	0.20	Primary + exploratory
Yilmaz 2021	Shannon/Hill	Diversity/evenness	−0.32	Primary + exploratory

Study-level standardized mean changes (Hedges’ g) in alpha diversity after direct-acting antiviral therapy, grouped by the ecological dimension of the reported metric. The stratification is descriptive: no pooled subgroup estimate was computed because the primary conservative set contains a single richness report (see Figure S2). Chuaypen 2021 contributes only to the exploratory five-report set.

Table S4. Quantitative extraction sheet used for the meta-analysis

Study	Metric	n	Data source	Pre mean	Pre SD	Post mean	Post SD	r	Weight % [r=0.50]
Chuaypen 2021	Chao1	81	Figure-assisted (boxplot)	100.0	23.0	140.0	39.0	0.50	20.5
Chuaypen 2023	Chao1	66	Text mean ± SD (responders)	99.91	40.05	128.50	46.50	0.50	20.6
Hsu 2022	Shannon	42	Figure-assisted (boxplot)	4.83	0.53	4.84	0.47	0.50	20.4
Honda 2021	Shannon	14	Figure-assisted (Pre vs Post24)	1.95	0.45	2.05	0.47	0.50	17.8
Yilmaz 2021	Hill (effective species, q=1)	58	Figure-assisted (bar plot)	72.0	25.0	64.0	25.0	0.50	20.7

Within-subject correlation sensitivity (five-report exploratory REML + Hartung–Knapp): $r = 0.25 \rightarrow g = 0.30$ (95% CI −0.32 to 0.91), $\tau^2 = 0.22$; $r = 0.50 \rightarrow g = 0.35$ (95% CI −0.38 to 1.07), $\tau^2 = 0.32$; $r = 0.75 \rightarrow g = 0.45$ (95% CI −0.49 to 1.40), $\tau^2 = 0.56$.

Note on Chuaypen cohort accounting: Chuaypen 2023 enrolled 69 participants, but the quantitative extraction used n = 66 for the responder-based longitudinal analysis; this explains the difference between Table S4 and Table S5. Note on cohort independence: Chuaypen 2021 and Chuaypen 2023 were generated by the same Thai research group, and participant overlap could not be excluded from published information; the main manuscript therefore uses a conservative non-overlap analysis and retains the five-report analysis as exploratory.

n, number of paired observations; SD, standard deviation; r, assumed within-subject pre/post correlation; REML, restricted maximum likelihood; Hartung–Knapp, Hartung–Knapp confidence-interval adjustment.

Table S5. Characteristics of included studies

Study	Country	Design	Cohort accounting	Fibrosis	DAA regimen	Alpha-diversity metric	Main findings	NOS
Elsherbiny 2025	Egypt	Cross-sectional	20 treated, 20 non-treated, 20 relapsed, 20 healthy controls	Non-cirrhotic (<7 kPa)	SOF/DCV	Shannon; observed species	Treated group closer to control; relapsed group had lowest diversity	7
Chuaypen 2023	Thailand	Longitudinal	69 enrolled (50 HCV mono, 19 HCV/HIV); responder-based longitudinal analyses at FU week 72	Mixed fibrosis	Various DAA regimens	Chao1; Simpson	Long-term improvement mainly in responders and lower-fibrosis strata; lower LBP after treatment	8
Inoue 2025	Japan	Longitudinal + cross-sectional	49 longitudinal (29 CH, 20 LC/HCC); cross-sectional CH-HCV 95, LC/HCC-HCV 79, CH-SVR 29, LC/HCC-SVR 46, healthy 23	CH to LC/HCC	Various guideline-based DAA regimens	Shannon–Wiener	Post-SVR groups shifted toward healthy profiles; bile-acid remodeling accompanied microbiota recovery	8
Hsu 2022	Taiwan	Longitudinal + matched control	42 HCV; 84 matched controls	Mixed fibrosis/cirrhosis	SOF-based regimens and GLE/PIB	Observed; Chao1; Shannon; Simpson	Overall alpha diversity did not significantly change at 12 weeks off therapy	7
Honda 2021	Japan	Longitudinal	14 patients sampled at Pre, EOT, and Post24	Mixed fibrosis	EBR/GZR, OBV/PTV/r, SOF/LDV, GLE/PIB	Chao1; Observed; Shannon	No overall diversity change; Faecalibacterium and Bacillus increased at Post24	6
Yilmaz 2021	Switzerland	Longitudinal	58 patients	Mixed fibrosis and genotypes 1–6	GLE/PIB	Species richness; Shannon; Simpson	No statistically significant alpha- or beta-diversity improvement after treatment	7
Chuaypen 2021	Thailand	Longitudinal	86 enrolled (62 HCV mono, 24 HCV/HIV); 81 with post-treatment stool	Mixed fibrosis	EBR/GZR	Chao1; Shannon; Simpson	Short-term restoration most evident in responders and in lower-fibrosis strata	8

All included studies used 16S ribosomal RNA (16S rRNA) V3–V4 amplicon sequencing. CH, chronic hepatitis; LC, liver cirrhosis; HCC, hepatocellular carcinoma; DAA, direct-acting antiviral; SOF, sofosbuvir; DCV, daclatasvir; EBR, elbasvir; GZR, grazoprevir; OBV, ombitasvir; PTV, paritaprevir; r, ritonavir; GLE, glecaprevir; PIB, pibrentasvir; EOT, end of treatment; FU, follow-up; LBP, lipopolysaccharide-binding protein; NOS, Newcastle–Ottawa Scale; HCV, hepatitis C virus; HIV, human immunodeficiency virus; SVR, sustained virological response; SOF, sofosbuvir; DCV, daclatasvir; LDV, ledipasvir; EBR, elbasvir; GZR, grazoprevir; OBV, ombitasvir; PTV, paritaprevir; r, ritonavir; GLE, glecaprevir; PIB, pibrentasvir.

Table S6. Leave-one-out sensitivity (five-report exploratory set)

Analysis	Pooled Hedges' g	95% CI	τ^2	Interpretation
Omit Chuaypen 2021 (= primary conservative)	0.13	−0.53 to 0.80	0.15	Direction attenuated; still inconclusive
Omit Chuaypen 2023	0.27	−0.76 to 1.30	0.40	Still inconclusive
Omit Hsu 2022	0.43	−0.59 to 1.45	0.39	Still inconclusive
Omit Honda 2021	0.38	−0.67 to 1.43	0.42	Still inconclusive
Omit Yilmaz 2021	0.52	−0.30 to 1.35	0.24	Still inconclusive

Checklist S1. Minimum Reporting Checklist for Future HCV Microbiome Recovery Studies

Proposed as a domain-specific companion to community reporting standards (e.g., STORMS for microbiome studies and MIxS for sequence metadata). Authors can copy the checkbox column into their own submissions to document compliance.

Reported (✓/✗)	Item	Recommended reporting	Rationale
☐	Sampling time	Baseline, end of treatment, SVR12, SVR24, and preferably SVR48	Allows trajectory-based interpretation of recovery
☐	Sequencing method	16S target region or shotgun metagenomics platform and bioinformatic pipeline	Improves comparability and reproducibility
☐	Diversity metrics	Shannon, Chao1/observed features, and a beta-diversity distance metric	Supports future quantitative synthesis
☐	Effect-size inputs	Per-timepoint mean, SD, and n (or paired change and pre/post correlation)	Enables standardized mean-change pooling without figure reconstruction
☐	Medication exposures	Antibiotics, probiotics, prebiotics, PPIs, and metformin exposure	Controls major microbiome confounders
☐	Host factors	Fibrosis stage, diabetes, BMI, alcohol use, HIV status, and diet	Prespecified effect modifiers; helps explain heterogeneity
☐	Functional profiling	Shotgun metagenomics, LC-MS metabolomics, and bile-acid profiling where feasible	Links composition to function
☐	Data sharing	Deposit raw sequence data and analysis code in public repositories	Enables verification and future meta-analysis

SVR, sustained virological response; PPI, proton-pump inhibitor; BMI, body mass index; HIV, human immunodeficiency virus; LC–MS, liquid chromatography–mass spectrometry.

All meta-analyses were conducted using R version 4.6.0 (R Foundation for Statistical Computing, Vienna, Austria) with the metafor package version 5.0-1.

Primary versus exploratory pooled estimates

Under the five-report exploratory REML + Hartung–Knapp framework, the pooled standardized mean change was Hedges' $g = 0.35$ (95% CI −0.38 to 1.07; $\tau^2 = 0.32$), with an approximate prediction interval of −1.3 to 2.0. Because potential participant overlap between Chuaypen 2021 and Chuaypen 2023 could not be excluded, the conservative non-overlap interpretation retained Chuaypen 2023 (the longer-follow-up report) and omitted Chuaypen 2021 from the primary analysis; this attenuated the pooled estimate to $g = 0.13$ (95% CI −0.53 to 0.80; $\tau^2 = 0.15$; $I^2 \approx 89\%$; $Q = 26.3$, $df = 3$, $p < 0.001$).

Metric-stratified (richness vs diversity/evenness) analysis

Because Chao1 (richness) and Shannon/Hill-type (diversity/evenness) indices capture distinct ecological dimensions, a metric-stratified descriptive analysis was pre-specified as a limitation of cross-metric pooling. The richness stratum was positive (Chuaypen 2023 Chao1 $g = 0.65$; Chuaypen 2021 Chao1 $g \approx 1.17$ in the exploratory set), whereas the Shannon/Hill-type diversity estimates from Hsu 2022, Honda 2021, and Yilmaz 2021 were individually near null ($g = 0.02$, 0.20, and −0.32, respectively). Because only a single report contributed richness data in the conservative set and only three reports contributed comparable diversity/evenness data, no pooled subgroup diamond was computed; the stratification is presented descriptively (Figure S2).

Supplementary Figures

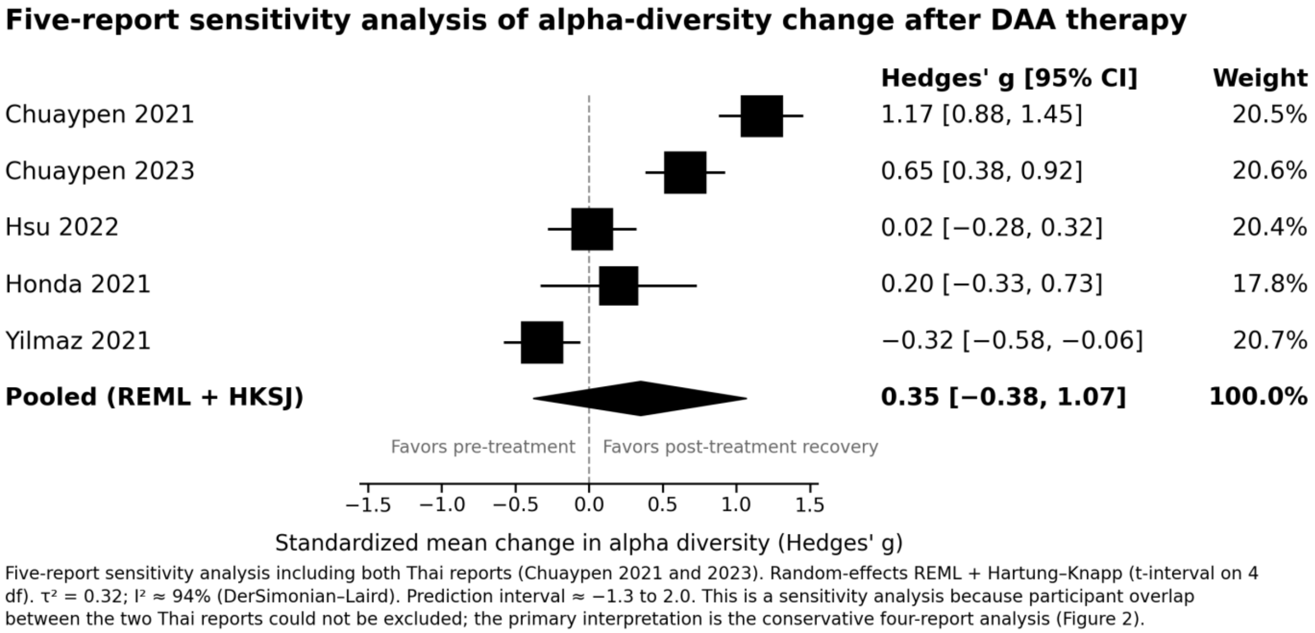

Figure S1. Five-report sensitivity analysis of longitudinal alpha-diversity change after direct-acting antiviral (DAA) therapy. This analysis includes both Thai reports (Chuaypen 2021 and 2023) and is retained as a sensitivity analysis because participant overlap could not be excluded. Random-effects pooling used REML with Hartung–Knapp adjustment. The pooled Hedges' g was 0.35 (95% CI −0.38 to 1.07; $\tau^2 = 0.32$), with an approximate prediction interval of −1.3 to 2.0; the primary interpretation is the conservative four-report non-overlap analysis shown in Figure 2.

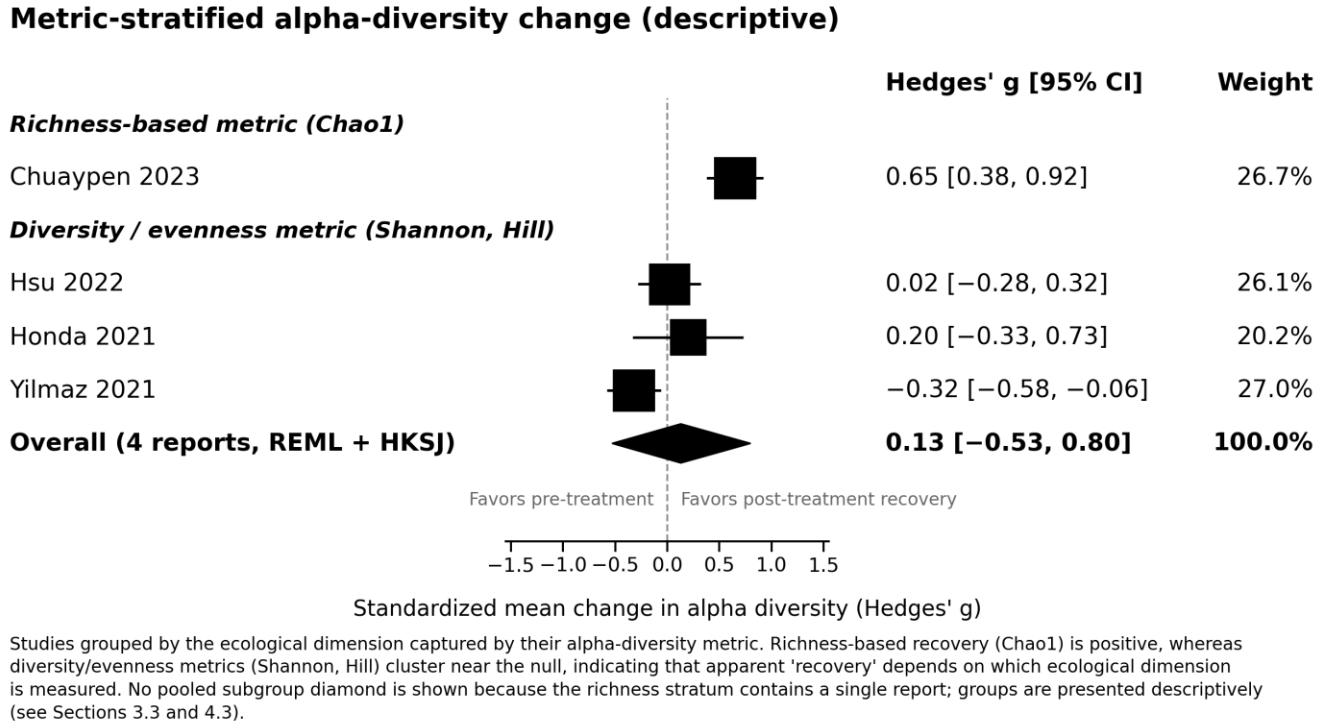

Figure S2. Metric-stratified alpha-diversity change after DAA therapy (descriptive). Studies are grouped by the ecological dimension captured by their alpha-diversity metric. The richness-based Chao1 estimate is positive, whereas Shannon/Hill diversity or evenness estimates cluster near the null. No pooled subgroup diamond is shown because the richness stratum contains a single report; the groups are presented descriptively (see Sections 3.3 and 4.3 and Supplementary Table S3).
